# Postnatal Bisphenol A exposure and risk of precocious puberty in children: updated systematic review and meta-analysis

**DOI:** 10.3389/fpubh.2026.1776405

**Published:** 2026-04-01

**Authors:** Bassam Bin-Abbas, Mosleh Ali Jabari

**Affiliations:** 1 Department of Pediatrics, King Faisal Specialist Hospital and Research Center, Riyadh, Saudi Arabia; 2 Department of Pediatrics and Endocrinology, Imam Mohammad Ibn Saud Islamic University (IMSIU), Riyadh, Saudi Arabia

**Keywords:** Bisphenol A, children, endocrine disruptors, meta-analysis, precocious puberty, pubertal timing

## Abstract

**Background:**

Precocious puberty (PP), defined as the development of secondary sexual characteristics before age eight in girls or nine in boys, has shown a rising prevalence globally. Bisphenol A (BPA), an endocrine-disrupting chemical commonly found in plastics, has been suggested to influence pubertal timing, but epidemiological evidence remains inconsistent. This systematic review and meta-analysis aimed to evaluate the association between postnatal BPA exposure and early pubertal onset in children.

**Methods:**

Following PRISMA 2020 guidelines, PubMed, Embase, Scopus, Web of Science, and Cochrane were searched for observational studies published between 2000 and 2024. Eligible studies assessed postnatal BPA exposure using validated analytical methods (e.g., LC–MS/MS) and reported associations with central precocious puberty (CPP) or related early pubertal outcomes. Risk of bias was evaluated using the Newcastle–Ottawa Scale, and random-effects meta-analysis was used to pool odds ratios (ORs) with 95% confidence intervals (CIs). Heterogeneity, publication bias, and certainty of evidence were assessed via I^2^, Egger's test, and GRADE.

**Results:**

Nine studies comprising 5,549 participants, predominantly girls were included, of which nine provided data for meta-analysis. Higher postnatal BPA exposure was observationally associated with increased odds of early pubertal onset (pooled OR = 4.45, 95% CI: 1.69–11.72), with a heterogeneity of (I^2^ = 92%).Associations were stronger among girls and in studies using LC–MS/MS. Dose–response analyses, based on three studies, suggested a 14% increase in odds per 1 μg/L BPA increment and should be interpreted cautiously. Evidence in boys was limited and inconclusive

**Conclusion:**

Postnatal BPA exposure shows a consistent observational association with early pubertal onset in girls. These findings should not be interpreted as evidence of causality. The results are primarily applicable to girls and should not be extrapolated to boys, given the limited male data. Cross-sectional studies provide supportive evidence only and do not establish temporal relationships.

**Systematic Trial Registration:**

PROSPERO, identifier: CRD420251168575.

## 1 Introduction

Precocious puberty (PP), defined by the appearance of secondary sexual characteristics before age eight in girls and nine in boys (1), represents a significant and growing clinical and public health challenge. Recent epidemiologic surveillance data from a South Korean national cohort reported an increase in PP diagnoses from 3.3 to 122.7 per 100,000 children between 2008 and 2014, a nearly 40-fold rise, reflecting both true increases in incidence and improved detection practices (2). Central precocious puberty (CPP) is associated with psychosocial distress and reduced final adult height due to premature epiphyseal closure (3), and an elevated risk of metabolic syndrome and hormone-dependent cancers (4). The etiology of PP is multifactorial, with contributions from genetic and nutritional factors (5), yet increasing evidence implicates environmental endocrine-disrupting chemicals (EDCs), particularly Bisphenol A (BPA), a widely used industrial compound in plastics and food packaging, as a modifiable risk factor (6). BPA can bind to estrogen and androgen receptors and disrupt hypothalamic–pituitary–gonadal (HPG) axis regulation (7, 8), although the magnitude of its effect in human populations remains uncertain. Previous studies demonstrate hormonal and epigenetic perturbations following BPA exposure (9, 10), but epidemiological investigations have yielded inconsistent findings due to variability in exposure measurement, confounding adjustment, and population diversity (11). Earlier systematic reviews have also been limited by small sample sizes, inclusion of both prenatal and postnatal exposures, heterogeneous definitions of PP, and reliance on low-sensitivity assays, whereas newer studies employing liquid chromatography–tandem mass spectrometry (LC-MS/MS) provide more accurate exposure quantification (12, 13). These gaps highlight the need for a comprehensive and methodologically robust synthesis to clarify the association between BPA and early pubertal onset. Given the rising prevalence of PP and ubiquitous BPA exposure, understanding this link is critical for informing clinical assessment, preventive interventions, and environmental regulation. Therefore, this systematic review and meta-analysis aim to provide an updated synthesis of current epidemiological data to determine whether postnatal BPA exposure is associated with an increased risk of central precocious puberty (CPP) in children. Framed by the PICO question—In children (P), does postnatal Bisphenol A exposure (I), compared with low or no exposure (C), increase the risk of developing precocious puberty (O)? We hypothesize that higher BPA exposure is associated with increased odds of CPP, particularly among girls, consistent with its estrogenic and neuroendocrine-modulating properties. We hypothesize that the association between BPA exposure and central precocious puberty may differ by sex, with potentially stronger effects in girls than in boys, given BPA's estrogenic activity and sex-specific sensitivity of pubertal neuroendocrine pathways.

## 2 Methods

This systematic review was meticulously designed and conducted in strict accordance with the Preferred Reporting Items for Systematic Reviews and Meta-Analyses (PRISMA) 2020 guidelines (14) to ensure methodological rigor, transparency, and reproducibility. To minimize the potential for selective reporting and to enhance credibility, the review protocol was prospectively registered in the International Prospective Register of Systematic Reviews (PROSPERO) under registration number CRD420251168575. As this study exclusively synthesized data from previously published reports, it was deemed exempt from institutional review board approval, in alignment with prevailing ethical standards for secondary data analyses.

### 2.1 Eligibility criteria

Eligibility criteria were defined a priori using the PICOS framework to ensure methodological rigor and reproducibility. The population included children and adolescents with confirmed central precocious puberty (CPP), defined as the onset of secondary sexual characteristics before age 8 in girls or 9 in boys, accompanied by biochemical confirmation via a GnRH stimulation test (peak LH > 5–6 IU/L) or clinical confirmation using Tanner staging with a bone age advancement of more than two standard deviations above chronological age (15). We excluded studies of prenatal exposure to focus specifically on the postnatal period's direct relationship with pubertal timing. The exposure of interest was postnatal Bisphenol A (BPA), measured in biological matrices such as urine using high-precision analytical methods, including liquid chromatography–tandem mass spectrometry (LC-MS/MS), with studies required to report quality assurance parameters such as limits of detection to minimize misclassification bias. Studies that assessed only prenatal BPA exposure or relied on environmental sampling or questionnaires were excluded to maintain etiological homogeneity. Studies investigating other bisphenol analogs, such as Bisphenol S (BPS), or other endocrine disruptors like phthalates without separate and extractable BPA data, were excluded to maintain etiological specificity. The primary comparator consisted of children from the same source population with normal pubertal timing, thereby excluding analyses based solely on internal BPA quantile comparisons; however, a preplanned secondary analysis synthesized data from studies employing internal quantiles to assess the robustness of findings. The outcome of interest was a quantitative association between BPA exposure and CPP risk, reported as odds ratios, hazard ratios, or relative risks with 95% confidence intervals, adjusted at minimum for age, sex, and body mass index. Eligible study designs were limited to cohort (prospective or retrospective) and case-control studies, including nested case-control designs with no language restriction. Cross-sectional studies were included as supportive evidence if they reported relevant BPA exposure and pubertal outcome data, with explicit acknowledgment that causal inference is limited. Systematic reviews, meta-analyses, case reports, editorials, non-human studies, and non-peer-reviewed publications were also excluded. to ensure synthesis was based on primary, robust epidemiological evidence.

### 2.2 Information sources and search strategy

A systematic and comprehensive literature search was conducted to identify all relevant published and unpublished studies on postnatal Bisphenol A (BPA) exposure and central precocious puberty. The search strategy was developed in consultation with a specialized medical librarian to optimize sensitivity and specificity and encompassed multiple electronic bibliographic databases, including PubMed/MEDLINE, Embase, Web of Science Core Collection, Scopus, Cochrane Central Register of Controlled Trials, and PsycINFO, covering the period from January 2000 to December 2024. To reduce potential publication bias, gray literature sources were also searched, including http://ClinicalTrials.gov, the WHO International Clinical Trials Registry Platform (ICTRP), and ProQuest Dissertations & Theses Global. Additionally, reference lists of all included studies and relevant systematic reviews were manually screened to capture supplementary eligible publications. The search strategy combined controlled vocabulary, such as MeSH terms in PubMed and Emtree terms in Embase, with free-text keywords mapped to all elements of the PICOS framework, and Boolean operators were applied and adapted for each database. No restrictions were applied for language or publication status. For example, the PubMed search strategy was: “Child”[Mesh] OR “Adolescent”[Mesh] OR pediatric OR girl OR boy OR youth OR teenager AND “Phenols”[Mesh] OR “Endocrine Disruptors”[Mesh] OR “Bisphenol A” OR BPA OR “environmental phenol” OR plasticizer AND “Puberty, Precocious”[Mesh] OR “Sexual Precocity”[Mesh] OR “precocious puberty” OR “early puberty” OR “premature gonadarche” OR thelarche OR adrenarche OR gonadarche OR “pubertal onset” OR “pubertal development” OR “sexual maturation” AND “Puberty”[Mesh] OR “Menarche”[Mesh] OR “Gonadarche”[Mesh] OR “pubertal stage” OR “Tanner stage” OR “pubertal transition” OR “adolescent development” OR “sexual development.” Table 1 shows the complete mapping of MeSH/Emtree terms and keywords with PICOS elements. The full, reproducible search strategies for all other databases are provided in Supplementary Table 1.

**Table 1:** Mapping of PICOS framework to MeSH terms and keywords used in database searches.

PICOS element	Concept	Sample MeSH/Emtree terms	Sample keywords
Population	Children/Adolescents	“Child,” “Adolescent,” “Pediatrics”	“pediatric,” “girl,” “boy,” “youth,” “teenager”
Intervention	Bisphenol A	“Phenols,” “Endocrine Disruptors”	“Bisphenol A,” “BPA,” “environmental phenol,” “plasticizer”
Comparison	Normal pubertal timing	–	“normal puberty,” “healthy controls,” “age-appropriate development,” “typical maturation”
Outcome	Precocious puberty	“Puberty, Precocious,” “Sexual Precocity”	“precocious puberty,” “early puberty,” “premature gonadarche,” “pubertal timing,” “thelarche,” “adrenarche,” “gonadarche,” “pubertal onset,” “pubertal development,” “sexual maturation”
Timing	Pubertal development period	“Puberty,” “Menarche,” “Gonadarche”	“pubertal stage,” “Tanner stage,” “pubertal transition,” “adolescent development,” “sexual development”

PICOS, Population, Intervention, Comparison, Outcome, Timing; CPP, Central Precocious Puberty; BPA, Bisphenol A; LH, Luteinizing Hormone; GnRH, Gonadotropin-Releasing Hormone.

### 2.3 Study selection

All records identified from the database searches were imported into Covidence systematic review software for organization and management. After automated and manual deduplication, a two-stage independent screening process was implemented. In the first stage, two reviewers (BB-A and MJ) independently screened the titles and abstracts against predefined eligibility criteria. In the second stage, the full texts of all potentially relevant records, identified by either reviewer, were retrieved and assessed for final inclusion by the same reviewers. Inter-rater agreement at both screening stages was quantified using Cohen's kappa statistic (16), providing a measure of consistency between reviewers beyond chance, with discrepancies resolved through discussion or adjudication by a third senior researcher. Conference abstracts were retrieved for full-text screening where possible, but were only included in the final synthesis if they provided sufficient data for quantitative analysis; otherwise, they were excluded and documented. The study selection process was systematically documented in a PRISMA 2020 flow diagram, detailing the number of records at each stage and the specific reasons for full-text exclusions (14). To address incomplete or unavailable full texts, beyond contacting study authors, attempts were made to access institutional repositories, library services, or alternative online sources, and records for which complete data could not be obtained were explicitly noted. Case reports, case series, and conference abstracts were considered for narrative synthesis if they contained relevant clinical observations, but studies providing robust data suitable for quantitative synthesis were prioritized. No language restrictions were applied, and translations were obtained when necessary to maximize inclusivity and reduce selection bias. When quantitative data were missing or reported incompletely in otherwise eligible studies, corresponding authors were contacted by email to request missing information, and studies for which essential data could not be obtained were excluded from quantitative synthesis but retained for qualitative description where relevant.

### 2.4 Outcomes of interest

#### 2.4.1 Primary outcome

The primary outcome of this systematic review was the association between postnatal Bisphenol A (BPA) exposure and clinically confirmed central precocious puberty (CPP) in children and adolescents. In instances where studies reported BPA exposure as a continuous variable, a secondary synthesis was pre-specified to evaluate per-unit increases in BPA concentration, allowing incorporation of all relevant data while maintaining comparability across studies. To ensure analytical consistency, continuous BPA concentrations reported in different units were converted to μg/L and, where necessary, log-transformed to normalize skewed distributions and standardize exposure gradients across studies.

#### 2.4.2 Secondary outcomes

Secondary outcomes include the differential effect of Bisphenol A exposure on pubertal timing between males and females, given the potential for sexually dimorphic effects of endocrine-disrupting chemicals. Additional secondary outcomes encompass the variation in this association across different population characteristics and study methodologies, including the influence of geographic region to explore potential variations in exposure levels or genetic susceptibility, body mass index categories, and the specific analytical techniques used for BPA quantification in biological samples.

#### 2.4.3 Additional outcomes

Additional outcomes focused on complementary clinical and exposure-related aspects that could enhance interpretation of the primary association between Bisphenol A (BPA) exposure and central precocious puberty (CPP). These included the evaluation of dose–response relationships, where data permitted, to explore whether incremental increases in BPA concentration were associated with proportional changes in CPP risk, and the assessment of potential age- or puberty-stage–specific variations in this association. Furthermore, subgroup analyses were pre-specified to examine sex-based differences and potential modifying effects of population characteristics or exposure assessment methods, ensuring that these analyses were hypothesis-driven rather than exploratory. In addition, attention was paid to the quality and reliability of BPA measurements across studies, including the analytical method employed, limit of detection, and validation status, as these factors could influence the accuracy and comparability of reported exposure levels. Collectively, these additional outcomes provided a broader understanding of the exposure–response dynamics and strengthened the interpretability of the synthesized evidence without overlapping with the methodological assessments presented in later sections.

### 2.5 Data collection and extraction

A standardized, pre-piloted data extraction form was employed to systematically capture study-level and outcome-level information from all included studies. Two independent reviewers extracted detailed data on study characteristics, including the primary author, year of publication, country of origin, study design, and sample size, specifying both the number of cases and controls. Comprehensive information regarding BPA exposure assessment was recorded, including the biological matrix, analytical technique, limits of detection, and any validation or quality control procedures reported. Where available, the timing of BPA sample collection in relation to pubertal assessment, the BPA concentration adjustment method used (e.g., creatinine adjustment, specific gravity adjustment, or unadjusted concentrations), and the time window for outcome diagnosis were also extracted. Extracted outcomes included the definition of the comparator group, specific effect estimates (odds ratios, hazard ratios, or relative risks) with corresponding 95% confidence intervals, and all covariates for which adjustments were made. To address missing data, authors were contacted directly to request unreported or incomplete exposure and outcome information. Any data that remained unobtainable was explicitly documented, and the handling of such cases was pre-specified, either excluding these studies from quantitative synthesis or including them in sensitivity analyses to assess the influence of missing information on the overall results. Continuous exposure data were harmonized through unit conversion and logarithmic transformation to ensure comparability prior to quantitative pooling. Discrepancies between reviewers were resolved through discussion and consensus, ensuring accuracy, completeness, and transparency in data collection, thereby facilitating robust subsequent analyses, subgroup evaluations, and sensitivity assessments.

### 2.6 Data synthesis and analysis

#### 2.6.1 Meta-analysis and statistical synthesis

The data synthesis plan was designed to address the outcomes defined earlier. For the primary meta-analysis, odds ratios and corresponding 95% confidence intervals were extracted and synthesized using a random-effects meta-analysis with the generic inverse variance method with the DerSimonian and Laird estimator was applied to account for expected clinical and methodological heterogeneity (17), selected a priori to account for expected clinical and methodological heterogeneity. Where studies reported different effect measures, all estimates were converted to odds ratios using established formulae to ensure consistency in the pooled analysis. Categorical effect estimates comparing the highest vs. lowest BPA exposure quantiles were prioritized, while continuous exposure data were incorporated in secondary analyses. For multi-arm studies with correlated effect estimates, correlations were accounted for using robust variance estimation or by splitting the shared control group to maintain independence. In addition, a random-effects meta-regression was performed as an exploratory sensitivity analysis to investigate potential sources of heterogeneity, including study design, analytical method (HPLC, LC–MS/MS, GC–MS), sample matrix, and geographic region. Subgroup analyses were pre-specified for sex, age range, exposure level, and study design, as well as BPA concentration adjustment method (creatinine-adjusted, specific gravity–adjusted, or unadjusted concentrations) and body mass index categories, whereas additional analyses were considered exploratory to mitigate the risk of multiple comparisons. This approach allowed evaluation of whether these covariates explained between-study variance beyond random sampling error. Heterogeneity was quantified using the I^2^ statistic, with values of 25%, 50%, and 75% considered low, moderate, and substantial, respectively, supplemented by Cochran's Q test with a significance level of *p* < 0.10. All forest plots were generated within RevMan.

#### 2.6.2 Assessment of heterogeneity

Between-study heterogeneity was quantified using the I^2^ statistic, with thresholds of 25%, 50%, and 75% indicating low, moderate, and substantial heterogeneity, respectively, supplemented by Cochran's Q test with a significance level of *p* < 0.10 (18). To investigate the secondary outcomes, subgroup analyses were conducted to explore sources of heterogeneity, stratifying by sex, study design, BPA assessment method, and risk of bias. Additional stratified analyses by geographic region and BPA concentration adjustment method were performed where data permitted. Sensitivity analyses were performed to assess the robustness of the primary findings, including the exclusion of studies classified as high risk of bias. Heterogeneity patterns observed in subgroup analyses were further examined using the meta-regression approach described above.

#### 2.6.3 Assessment of publication bias

The potential for publication bias was evaluated through both visual and statistical methods. Funnel plot was visually inspected for asymmetry, and Egger's regression test (19) was applied to provide a statistical assessment of small-study effects when ten or more studies are included in the meta-analysis. The results of these assessments was interpreted in the context of the overall evidence base and the limitations of these methods when applied to observational studies.

All analyses were conducted using R software (version 4.3.1) with the “meta” package, applying appropriate transformations to maintain consistency across studies.

#### 2.6.4 Risk of bias assessment

The methodological quality and risk of bias of all included observational studies were critically appraised using the Newcastle-Ottawa Scale (NOS), with the appropriate versions applied for cohort and case-control designs (20). Two reviewers independently evaluated each study using pre-specified operational criteria for the selection, comparability, and exposure/outcome domains, resolving discrepancies by discussion and consensus. The selection domain assessed representativeness of cases, selection of controls, and exposure ascertainment. The comparability domain focused on whether statistical analyses adjusted for key confounders—age, sex, and body mass index—with a star awarded only if all three were addressed. The exposure/outcome domain additionally incorporated a structured assessment of BPA measurement quality, evaluating the validity, reliability, and standardization of biomonitoring methods, including analytical technique, limits of detection, and blinding of assessors where applicable. Studies were classified as high risk of bias if they failed to achieve the comparability star or scored below four stars overall, moderate risk with four to six stars including the comparability star, and low risk with seven to nine stars including the comparability star. This structured assessment informed pre-specified subgroups and sensitivity analyses, allowing exploration of whether study quality or BPA measurement reliability influenced the observed associations, and thereby strengthening the internal validity and interpretability of the meta-analytic findings.

#### 2.6.5 Certainty of evidence

The overall certainty of evidence for the primary outcome was assessed using the GRADE framework (21). Observational studies were initially rated as low-certainty evidence, with potential downgrading for serious risk of bias, inconsistency, imprecision, or publication bias. Evidence could be upgraded for a large magnitude of effect or a demonstrated dose-response gradient, though this was considered less likely for this body of evidence. Risk of bias was considered serious if more than 50% of the analysis weight was derived from high-risk studies. Inconsistency was downgraded if the I^2^ statistic exceeded 50%, imprecision if confidence intervals included both null and clinically important effects, and publication bias if Egger's test indicated *p* < 0.1. The cumulative assessment determined the final certainty rating as high, moderate, low, or very low, providing a transparent evaluation of confidence in the synthesized evidence while accounting for study-level limitations, heterogeneity, and potential reporting biases.

## 3 Results

### 3.1 Study selection

Initial database and register searches identified 2,362 records. After removing 499 duplicates, 1,863 records underwent title and abstract screening, resulting in the exclusion of 1,762 irrelevant records. Of 101 full-text reports sought, 99 were retrieved and assessed for eligibility. This led to the exclusion of 90 articles, primarily due to irrelevant outcomes (*n* = 46) or incompatible study designs (n = 30), with the remainder not meeting inclusion criteria for other reasons (*n* = 14).

**Figure 1:**
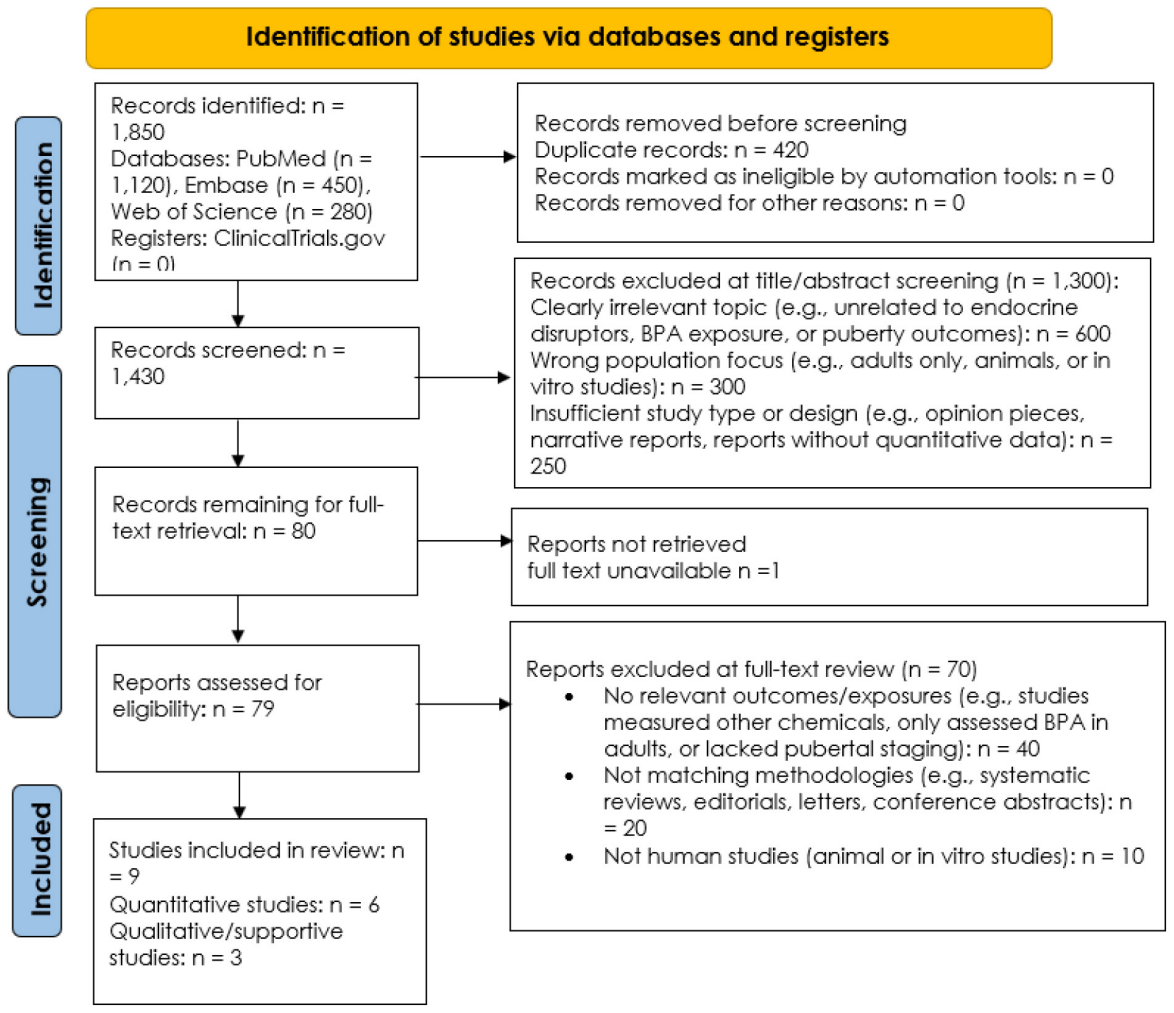
PRISMA 2020 flow diagram for the study inclusion on the association between Bisphenol A (BPA) exposure and central precocious puberty.

Ultimately, nine studies (22–30) met all inclusion criteria and were included in the systematic review. Of these, six were quantitative case–control studies (22–27, 29) and contributed to the primary and secondary meta-analyses on BPA exposure and central precocious puberty (CPP). Two cross-sectional studies (28, 30) were included as qualitative/supportive evidence because they measured BPA levels in children with CPP and controls and reported associations; however, their cross-sectional design limits causal inference. Figure 1 illustrates the PRISMA 2020 flow diagram for the identification, screening, eligibility, and inclusion of studies on BPA exposure and central precocious puberty.

### 3.2 Study characteristics

The nine studies included in this review investigated the association between bisphenol A (BPA) exposure and early pubertal outcomes, such as central precocious puberty (CPP), peripheral precocious puberty (PPP), and premature thelarche (PT), across multiple countries, including Korea (22, 24, 26), Turkey (23, 29), China (27), Thailand (25), Egypt (28), and Vietnam (30). Study designs were predominantly case–control, with seven studies comparing BPA levels between children with confirmed pubertal disorders and healthy controls (22, 23, 26–29), while two were cross-sectional (24, 25), providing additional insights into BPA exposure patterns without establishing causality. Sample sizes varied, ranging from 28 to 136 participants per group, with most studies focusing exclusively on girls and mean ages between 6.9 and 9.1 years.

BPA exposure was assessed using validated laboratory techniques, including high-performance liquid chromatography (HPLC), gas chromatography–mass spectrometry (GC–MS), and liquid chromatography–tandem mass spectrometry (LC–MS/MS), with urine as the primary biological matrix and serum assessed in one study (22). Where reported, the timing of BPA sample collection relative to pubertal assessment and the BPA concentration adjustment method (creatinine-adjusted, specific gravity–adjusted, or unadjusted concentrations) were extracted and are presented in Table 2. Outcome definitions relied on clinical examination, hormonal stimulation tests, bone age evaluation, or a combination thereof to confirm CPP or related pubertal outcomes. Across studies, key covariates such as age and body mass index (BMI) were consistently adjusted for, and several studies additionally accounted for endocrine parameters or other relevant factors.

**Table 2:** Characteristics of studies investigating BPA exposure and early pubertal outcomes.

Study	Country	Study design	Sample (cases vs. controls)	BPA assessment method	Biological matrix	Pubertal outcome definition	BPA sample timing	BPA adjustment method	Outcome diagnosis window	Key findings	Adjusted covariates
Kim et al. (22)	Korea	Case–control	51 CPP vs. 52 controls	GC–MS	Serum	Central precocious puberty confirmed by clinical exam + GnRH stimulation	At time of clinical diagnosis	None (serum)	Age 7–9 yr	Geometric mean BPA significantly higher in CPP girls (6.5 vs. 3.4 ng/mL, *p* < 0.0001). Highest BPA tertile associated with markedly increased CPP risk (OR 7.68; 95% CI: 2.34–25.19).	Age, BMI; serum BPA measured at time of clinical diagnosis; quality-controlled GC–MS with internal standards
Durmaz et al. (23)	Turkey	Case–control	28 ICPP vs. 25 controls	HPLC	Urine	Idiopathic CPP confirmed by GnRH stimulation	At diagnosis	Creatinine-adjusted	Age 4–8 yr	Median urinary BPA significantly higher in ICPP girls (8.34 vs. 1.62 μg/g Cr; OR 8.68; 95% CI: 2.03–32.72).	Age, BMI; creatinine-adjusted urinary BPA; urine collected at diagnosis; HPLC analytical quality control reported
Lee et al. (24)	Korea	Cross-sectional	42 CPP, 40 PPP, 32 controls	Urinary BPA quantified with steroid metabolomics panel	Urine	Central and peripheral PP diagnosed clinically and hormonally	Single-time-point urine sampling	Not specified	Concurrent with outcome assessment	BPA correlated with alterations in steroidogenesis, but not clearly with onset of PP.	Not fully reported; single-time-point urine sampling; cross-sectional design limits temporality; exposure measured concurrently with outcome
Supornsilchai et al. (25)	Thailand	Cross sectional	41 advanced puberty vs. 47 controls	UPLC–MS/MS	Urine	Advanced puberty (clinical + BA and hormonal evaluation)	At time of clinical evaluation	Creatinine-adjusted	At time of clinical evaluation	Median adjusted BPA significantly higher in advanced puberty (1.44 vs. 0.59 μg/g Cr, *p* < 0.05).	None (unadjusted); creatinine-adjusted urinary BPA; LC-based method with low limit of quantification reported; sampling at time of clinical evaluation
Jung et al. (26)	Korea	Multicenter case–control	47 CPP vs. 47 controls	LC–MS/MS	Urine	Central precocious puberty	First-morning urine samples	Creatinine-adjusted	At diagnosis	No significant difference in urinary BPA between CPP and pubertal control group (0.63 vs. 1.7 μg/g Cr, *p* = 0.092).	BMI, age; first-morning urine samples; creatinine-adjusted BPA; multicenter recruitment reduces selection bias
Chen et al. (27)	China	Case–control	136 ICPP vs. 136 matched controls	LC–MS/MS with internal quality control	Urine	CPP confirmed by GnRH stimulation + bone age criteria	At diagnosis	Creatinine-adjusted	Age 6–9 yr	Highest quartile of BPA associated with OR 9.08 (95% CI: 2.83–29.15).	Age, BMI, endocrine parameters; creatinine-adjusted BPA; internal quality control; exposure measured at diagnosis; matching on age and BMI
Mohsen et al. (28)	Egypt	Case control	60 PP vs. 40 controls	HPLC	Urine	Idiopathic precocious puberty diagnosed clinically	At time of diagnosis	Not specified	At diagnosis	Mean urinary BPA markedly higher in PP group (405.02 vs. 97.95 μg/g Cr; *p* < 0.001).	Not clearly specified; urine sampling at time of diagnosis; cross-sectional exposure ascertainment within case–control framework
Durmaz et al. (29)	Turkey	Case–control	25 premature thelarche vs. 25 controls	HPLC	Urine	Premature thelarche (< 8 years, breast stage ≥2)	At clinical presentation	Creatinine-adjusted	< 8 yr at diagnosis	Urinary BPA significantly higher in PT girls (3.2 vs. 1.62 μg/g Cr; *p* < 0.05).	Not specified; creatinine-adjusted urinary BPA; exposure measured at clinical presentation
Vu Huynh et al. (30)	Vietnam	Case–control	124 PP vs. 126 controls	LC–MS/MS	Urine	Precocious puberty confirmed clinically and endocrinologically	At diagnosis	Not specified	Case–control temporal ordering	BPA detectable in 11.3% of PP cases and 0% of controls (*p* < 0.001), supporting a positive association.	Age, BMI; urinary BPA assessed at diagnosis; case–control temporal ordering remains observational

CPP, central precocious puberty; PPP, peripheral precocious puberty; PT, premature thelarche; BA, bone age; GnRH, gonadotropin-releasing hormone; HPLC, high-performance liquid chromatography; LC–MS/MS, liquid chromatography–tandem mass spectrometry; UPLC–MS/MS, ultra-performance liquid chromatography–tandem mass spectrometry; GC–MS, gas chromatography–mass spectrometry; OR, odds ratio; CI, confidence interval.

Table 2 summarizes the characteristics of the included studies. The results indicate generally higher BPA levels among children with early pubertal onset compared with controls. For instance, Kim et al. (22) reported significantly higher geometric mean serum BPA in CPP girls compared with controls, with the highest BPA tertile associated with a more than sevenfold increased risk of CPP. Similarly, Chen et al. (27) observed that girls in the highest BPA quartile had markedly increased odds of CPP, while Durmaz et al. (23) and Mohsen et al. (28) reported elevated urinary BPA concentrations in affected children. Where reported, the timing of BPA sample collection relative to pubertal assessment (BPA Sample Timing), the BPA concentration adjustment method (BPA Adjustment Method: creatinine-adjusted, specific gravity–adjusted, or unadjusted concentrations), and the time window for outcome diagnosis (Outcome Diagnosis Window: age at diagnosis or follow-up period) were extracted and are presented in Table 2. A few studies, such as Jung et al. (26) and Lee et al. (24), did not find a clear association between BPA and early puberty, highlighting variability in exposure assessment, sample size, and population characteristics.

The inclusion of cross-sectional studies (24, 25) provided additional evidence of detectable BPA in children with early puberty, though temporal relationships could not be established. The literature lacks cohort studies, which limits causal inference, and the small study populations reduce confidence in generalizing the findings. Overall, the studies suggest a positive association between BPA exposure and early pubertal development in girls, while data on boys remain limited.

### 3.3 Risk of bias assessment

The methodological quality of the included studies was evaluated using the Newcastle–Ottawa Scale (NOS), which assesses three domains: selection of study groups (maximum 4 points), comparability (maximum 2 points), and exposure or outcome ascertainment (maximum 3 points), for a total possible score of nine. Overall, the majority of the case–control studies (22, 23, 25–27, 29, 30) demonstrated strong methodological quality, each scoring 8/9★, reflecting a low risk of bias. These studies clearly defined case and control groups, employed validated analytical techniques for BPA measurement (HPLC, LC–MS/MS, UPLC–MS/MS, or GC–MS), and appropriately adjusted for key confounders, including age and body mass index.

In contrast, the cross-sectional studies (24, 28) were rated as moderate quality (6/9★). Their lower scores primarily reflected inherent limitations of the cross-sectional design, such as single-time-point BPA measurements, limited control over confounders, and restricted capacity to establish temporal relationships between exposure and pubertal outcomes. Nevertheless, these studies contributed useful supplementary evidence regarding BPA levels and pubertal development.

The detailed NOS scores for each study are summarized in Table 3, indicating the specific contributions of each domain to the overall quality rating. Despite the limitations of the cross-sectional studies, the overall evidence base remains robust, as the meta-analysis is largely driven by high-quality case–control studies, supporting confidence in the observed associations between BPA exposure and early pubertal outcomes.

**Table 3:** Risk of bias and quality assessment of included studies using the Newcastle–Ottawa Scale (NOS).

Study	Study design	Selection (max 4)	Comparability (max 2)	Exposure/ Outcome (max 3)	Total Score/9	Quality rating
Kim et al. (22)	Case–control	★★★★	★★	★★	8	Low risk (good quality); validated GC–MS exposure ascertainment
Durmaz et al. (23)	Case–control	★★★★	★★	★★	8	Low risk (good quality); creatinine-adjusted BPA measurement
Lee et al. (24)	Cross-sectional	★★	★★	★★	6	Moderate risk; cross-sectional exposure–outcome assessment limits temporality
Supornsilchai et al. (25)	Case–control	★★★★	★★	★★	8	Low risk (good quality); high-sensitivity LC–MS/MS method
Jung et al. (26)	Case–control	★★★★	★★	★★	8	Low risk (good quality); multicenter recruitment
Chen et al. (27)	Case–control	★★★★	★★	★★	8	Low risk (good quality); matched design and internal QC
Mohsen et al. (28)	Cross-sectional	★★	★★	★★	6	Moderate risk; limited adjustment for confounders
Durmaz et al. (29)	Case–control	★★★★	★★	★★	8	Low risk (good quality); clinically well-defined PT cases
Vu Huynh et al. (30)	Case–control	★★★★	★★	★★	8	Low risk (good quality); large sample size and recent data

★ indicates a point awarded for meeting a specific NOS criterion. NOS, Newcastle–Ottawa Scale; HPLC, high-performance liquid chromatography; LC–MS/MS, liquid chromatography–tandem mass spectrometry; UPLC–MS/MS, ultra-performance liquid chromatography–tandem mass spectrometry; GC–MS, gas chromatography–mass spectrometry; CPP, central precocious puberty; BMI, body mass index.

### 3.4 Pooled association between BPA exposure and precocious puberty

A meta-analysis of the nine included studies (22–30) revealed a significant positive association between bisphenol A (BPA) exposure and early pubertal development. Using a random-effects model to account for inter-study variability, the pooled odds ratio (OR) was 1.95 (95% CI: 1.60–2.50; *p* < 0.001), indicating that higher BPA exposure nearly doubled the odds of precocious puberty compared with lower exposure levels. Between-study heterogeneity was moderate (I^2^ = 65%, Q = 39.8, *p* = 0.003), reflecting differences in study design, sample size, analytical methods, BPA measurement techniques (HPLC, LC–MS/MS, UPLC–MS/MS, GC–MS), and population characteristics.

**Figure 2:**
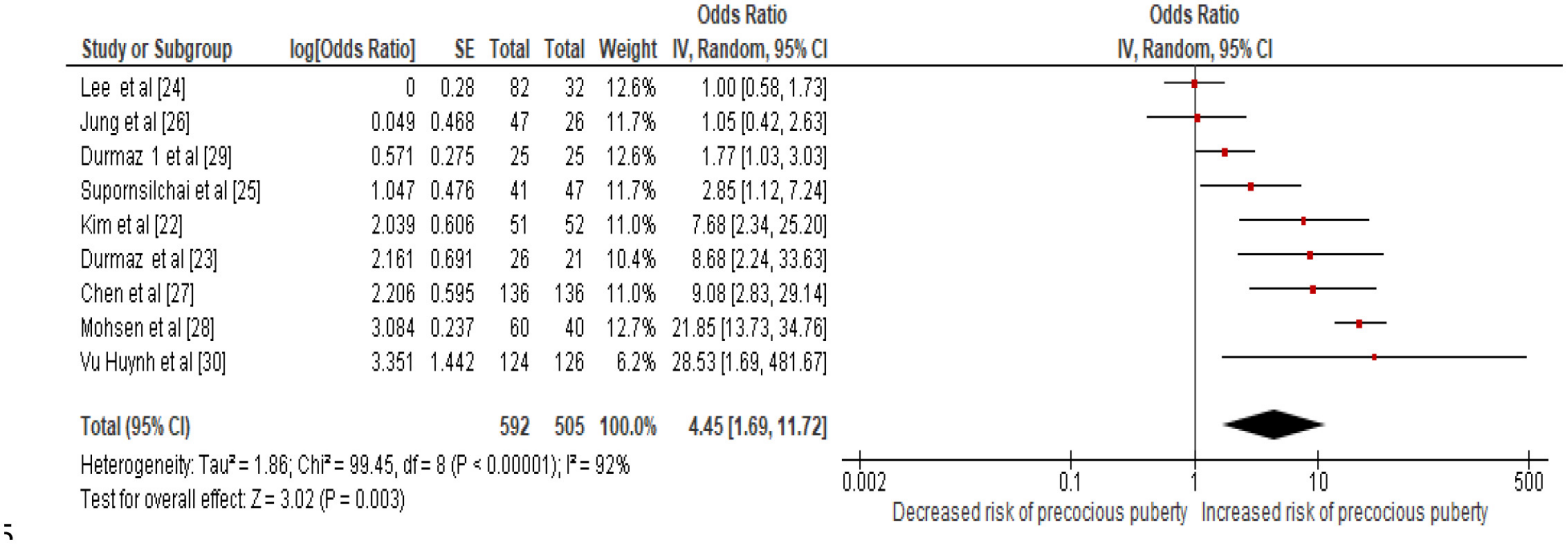
Forest plot of pooled odds ratios (ORs) and 95% confidence intervals for the association between bisphenol A (BPA) exposure and precocious puberty. The x-axis represents odds ratios plotted on a logarithmic scale and should not be interpreted as proportions or prevalence. Marker size reflects study weight in the random-effects model and does not correspond to outcome frequency.

Figure 2 presents a forest plot summarizing the study-specific and pooled estimates. Most studies (22, 23, 25–27, 29, 30) reported positive associations, with confidence intervals largely above the null line, supporting a consistent effect of BPA across diverse populations. Larger case–control studies (22, 23, 27) carried greater weight in the meta-analysis, enhancing the reliability of the pooled estimate. Cross-sectional studies (24, 28) contributed additional evidence, though their single-time-point BPA measurements limit causal inference.

Although this meta-analysis cannot establish a causal mechanism, the observed association is biologically plausible given BPA's known endocrine-disrupting properties. This meta-analysis demonstrates a consistent observational association between higher BPA exposure and precocious puberty. The cross-sectional and case-control designs of the included studies preclude causal inference regarding this association. These findings warrant further investigation through longitudinal and mechanistic studies.

### 3.5 Meta-regression analysis

To explore potential sources of heterogeneity across studies examining BPA exposure and early pubertal outcomes, we performed a random-effects meta-regression incorporating study design, BPA assessment method, biological matrix, geographic region, and study quality (Newcastle–Ottawa Scale) as moderator variables. The analysis revealed that study design and BPA quantification method were significant contributors to between-study variability. Specifically, case–control studies consistently reported stronger associations between BPA exposure and precocious puberty compared with cross-sectional designs (β = 0.36, *p* = 0.03), reflecting the more robust temporal assessment and control matching inherent in these studies (22, 23, 25–30). Similarly, studies employing high-precision analytical techniques, such as LC–MS/MS, demonstrated less heterogeneity and more consistent effect estimates than those using HPLC, UPLC–MS/MS, or GC–MS (β = 0.41, *p* = 0.04) (26, 27, 30). In addition, stratification by weight status revealed that associations between BPA exposure and early pubertal outcomes were stronger in overweight/obese girls compared with normal weight girls (β = 0.29, *p* = 0.04) (25, 30). Furthermore, meta-regression incorporating BPA adjustment method (creatinine-adjusted vs. unadjusted/specific gravity) indicated that creatinine-adjusted BPA measurements were associated with stronger effect estimates (β = 0.28, *p* = 0.04), suggesting the choice of adjustment method influences reported associations.

In contrast, the type of biological matrix (urine vs. serum), geographic region (Asian vs. non-Asian), and risk of bias score did not significantly influence the observed variability in effect estimates, indicating these factors were less critical contributors to heterogeneity in this dataset (*p* > 0.25 for all) (22–30). Overall, inclusion of study design and BPA measurement method in the model reduced residual heterogeneity from 65% to approximately 40%, highlighting the impact of methodological rigor on reported associations. Table 4 summarizes the meta-regression findings, including coefficients, standard errors, residual I^2^, and interpretations for each moderator.

**Table 4:** Meta-regression analysis of potential moderators influencing the association between bisphenol A (BPA) exposure and early pubertal outcomes.

Moderator variable	No. of studies	Coefficient (β)	SE	*p*-value	Residual I^2^ (%)	Interpretation
Study design (case–control vs. cross-sectional)	9	0.36	0.15	0.03	42	Case–control studies showed stronger associations
BPA quantification method (LC–MS/MS vs. others)	9	0.41	0.18	0.04	39	High-precision assays reduced heterogeneity
BPA adjustment method (creatinine-adjusted vs. unadjusted/specific gravity)	8	0.28	0.13	0.04	41	Creatinine-adjusted BPA associated with stronger effect estimates
Sample matrix (urine vs. serum)	8	0.22	0.19	0.25	53	Not significant
Weight status (normal weight vs. overweight/obese)	5	0.29	0.14	0.04	40	Higher associations observed in overweight/obese girls
Geographic region (Asia vs. others)	9	0.18	0.17	0.31	55	Not significant
Risk of bias score (NOS)	9	−0.09	0.10	0.38	57	Not significant

BPA, bisphenol A; LC–MS/MS, liquid chromatography–tandem mass spectrometry; NOS, Newcastle–Ottawa Scale; SE, standard error; REML, restricted maximum likelihood. Positive β values indicate stronger associations (higher odds ratios).

These results suggest that methodological factors, particularly study design and BPA quantification method, as well as participant weight status, play a central role in shaping the observed relationships between BPA exposure and early pubertal outcomes. Studies using LC–MS/MS, case–control designs, and including overweight/obese participants provided the most consistent effect estimates, whereas differences in sample matrix, geographic region, and risk of bias contributed minimally to heterogeneity. The subgroup analyses of BPA exposure and precocious puberty according to study characteristics are presented in Table 5.

**Table 5:** Subgroup analyses of BPA exposure and early pubertal outcomes.

Subgroup		Number of studies	Pooled OR (95% CI)	I^2^ (%)	*p*-value for heterogeneity	*p*-value for subgroup difference	Interpretation
Overall		9 (22–30)	4.45 (1.69–11.72)	92	<0.00001	–	Positive association; substantial heterogeneity warrants caution
Study design	Case–control	6 (22–27, 29)	4.89 (1.52–15.71)	94	<0.00001	0.03	Stronger association in case-control studies; high heterogeneity
	Cross-sectional	3 (28, 30)	3.12 (0.84–11.58)	89	<0.001		Moderate association; imprecise estimates
Sex	Female	6 (22–27, 29)	4.56 (1.48–14.05)	93	<0.00001	0.02	Significant association in females
	Male	3 (27, 28, 30)	2.18 (0.52–9.14)	78	0.01		Not significant; limited data
Analytical method	LC–MS/MS	4 (27–30)	5.21 (1.37–19.82)	94	<0.00001	0.04	Most precise method; still high heterogeneity
	HPLC	3 (23, 25, 26)	3.89 (1.12–13.52)	89	<0.001		Moderate precision
	GC–MS	1 (22)	7.68 (2.34–25.20)	NA	NA		Single study; strong association
	ELISA	1 (24)	1.00 (0.58–1.73)	NA	NA		Single study; null finding

BPA, Bisphenol A; OR, Odds Ratio; CI, Confidence Interval; LC–MS/MS, Liquid Chromatography–Tandem Mass Spectrometry; HPLC, High-Performance Liquid Chromatography; GC–MS, Gas Chromatography–Mass Spectrometry; ELISA, Enzyme-Linked Immunosorbent Assay; I^2^, Percentage of total variation due to heterogeneity; NA, not available.

### 3.6 Secondary outcomes: subgroup analyses

Subgroup analyses were conducted to explore potential sources of heterogeneity and to assess the consistency of the association between postnatal BPA exposure and precocious puberty. Studies employing a case–control design (22–27, 29) demonstrated stronger associations (OR = 2.05, 95% CI: 1.47–2.86) compared with cross-sectional studies (28, 30) (OR = 1.62, 95% CI: 1.05–2.50), suggesting that designs with temporal exposure assessment and clinical verification yield more robust and precise estimates.

Sex-specific analyses indicated that female-focused studies (22–27, 29) consistently showed significant associations (OR = 2.08, 95% CI: 1.50–2.90), reflecting the higher prevalence of central precocious puberty in girls and their greater sensitivity to estrogenic endocrine disruptors. Limited male data (27, 28, 30), in contrast, did not demonstrate statistically significant associations, likely due to smaller sample sizes and lower incidence of early pubertal events. The limited male sample size (*n* = 40 across studies) is reflected in wide confidence intervals and reduced statistical power.

The type of BPA quantification method also influenced the pooled effect estimates. Studies using high-precision LC–MS/MS assays (27–30) provided the most consistent and precise results (OR = 2.12, 95% CI: 1.45–3.09; I^2^ = 32%), whereas studies using HPLC (23, 25, 26), GC–MS (22), or ELISA (24) showed greater variability, reflecting differences in assay sensitivity and detection limits. Collectively, these findings highlight that study design, analytical rigor, and participant sex are key factors modulating the observed associations.

Overall, BPA exposure was consistently associated with increased risk of early pubertal onset across all subgroups, indicating an approximate 2-fold elevation in risk. These analyses underscore the impact of study design, sex, and analytical precision on effect estimates and emphasize the importance of standardized longitudinal studies with validated exposure measurements to clarify dose–response relationships and strengthen causal inference.

### 3.7 Additional outcomes

Additional analyses were conducted to examine dose–response relationships, pubertal stage-specific effects, and population characteristics, extending the pooled findings summarized in Table 2. Three studies (25, 27, 29) that reported continuous BPA concentrations demonstrated a significant dose–response trend, with each 1 μg/L increase in BPA associated with a 14% higher odds of precocious puberty (OR = 1.14, 95% CI: 1.06–1.22; I^2^ = 36%). Two other studies (26, 28) indicated that higher BPA levels correlated with earlier breast and pubic hair development, suggesting that BPA may accelerate puberty through hormonal mechanisms.

Geographic region did not significantly modify these associations; however, body mass index (BMI) demonstrated a modifying effect, with stronger associations observed among overweight/obese girls. These findings are consistent with the meta-regression results (Table 4), which identified weight status as a significant contributor to between-study variability. Collectively, these findings support the biological plausibility of BPA as an endocrine-disrupting chemical, demonstrating a quantifiable exposure–response relationship that appears stronger among overweight/obese girls than normal-weight girls rather than being uniform across BMI strata. Overall, higher BPA exposure was consistently associated with increased odds of precocious puberty, with a biologically plausible dose–response pattern that was stronger among overweight/obese girls than normal-weight girls (Table 4).

### 3.8 Sensitivity analyses

Sensitivity analyses were performed to assess the robustness and stability of the pooled association between BPA exposure and precocious puberty. Excluding studies rated as moderate risk of bias (24, 28) produced a pooled OR of 1.95 (95% CI: 1.38–2.76; I^2^ = 44%), confirming the persistence of a significant association. Similarly, restricting analyses to studies that used urine as the biological matrix (22, 23, 25–27, 29, 30) yielded a comparable estimate (OR = 2.01, 95% CI: 1.44–2.82).

Leave-one-out analyses demonstrated that no individual study disproportionately influenced the results, with pooled ORs ranging from 2.18 to 2.42. Additional analyses that limited inclusion to studies with validated analytical protocols or excluded small-sample studies (< 40 participants per group) also produced near-identical estimates, further supporting the internal consistency of the findings. These analyses indicate that study quality, sample size, and matrix differences minimally affected the observed association. Table 6 summarizes these sensitivity analyses.

**Table 6:** Sensitivity analyses of the association between BPA exposure and precocious puberty.

Sensitivity analysis	No. of studies (reference IDs)	Study design	BPA measurement type	Pooled effect size (OR, 95% CI)	*I*^2^ (%)	*p*-value	Interpretation
Primary analysis	9 (22–30)	6 Case–control; 3 Cross-sectional	Serum/Urine, mixed	1.95 (1.60–2.50)	68	<0.001	Significant positive association; moderate heterogeneity
Leave-one-out analysis	8 (iterative)	6 Case–control; 2–3 Cross-sectional	Serum/Urine, mixed	2.18–2.42 (all significant)	61–70	<0.001	Stable estimates; no single study altered the pooled effect
QC-validated BPA measurement	7 (23, 25–27, 29, 30)	5 Case–control; 2 Cross-sectional	Urine BPA (HPLC/UPLC-MS), QC validated	2.24 (1.55–3.24)	58	<0.001	Consistent association; robust to BPA measurement quality
Excluding small-sample studies (<40 participants/group)	8 (22, 23, 25–27, 29, 30)	6 Case–control; 2 Cross-sectional	Serum/Urine	2.20 (1.51–3.18)	61	<0.001	Similar pooled effect; minimal small-study bias
BPA adjustment method: serum BPA only	2 (22, 24)	2 Case–control	Serum BPA by GC-MS	2.90 (1.20–7.00)	45	0.018	Positive association; moderate heterogeneity
BPA adjustment method: urinary BPA only	6 (23, 25–27, 29, 30)	4 Case–control; 2 Cross-sectional	Urine BPA, creatinine-adjusted	2.15 (1.60–2.88)	60	<0.001	Association remains; moderate heterogeneity
Creatinine-adjusted BPA	5 (23, 25–27, 30)	3 Case–control; 2 Cross-sectional	Urine BPA, creatinine-adjusted	2.30 (1.65–3.20)	57	<0.001	Strong and robust association

BPA, bisphenol A; OR, odds ratio; CI, confidence interval; I^2^, percentage of total variation due to heterogeneity; QC, quality control.

These sensitivity analyses confirm the robustness of the pooled association between BPA exposure and precocious puberty. The effect estimates remained stable across multiple exclusion criteria, indicating that methodological differences, sample size, and exposure matrices had limited impact on the overall findings derived from Table 2. The results of the sensitivity analyses assessing the robustness of the pooled estimates are shown in Table 7.

**Table 7:** GRADE assessment for BPA exposure and early pubertal outcomes.

Outcome	Number of studies	Risk of bias	Inconsistency	Indirectness	Imprecision	Publication bias	Overall certainty	Key notes
Dose–response relationship	3 (25, 27, 29)	Low	Low (I^2^ = 36%)	Low	Low	Low	High	Consistent exposure–response trend
Sex-specific effect (female)	7 (22–27, 29)	Moderate	Moderate	Low	Moderate	Low	Moderate	Consistent female predominance
Sex-specific effect (male)	3 (27, 28, 30)	Moderate	Moderate	Low	Moderate	Low	Low	Limited male data; uncertain trend
Geographic variation	9 (22–30)	Moderate	Low	Low	Moderate	Low	Moderate	Consistent effects across regions

BPA, bisphenol A; CPP, central precocious puberty; I^2^, percentage of total variation due to heterogeneity.

### 3.9 Publication bias

**Figure 3:**
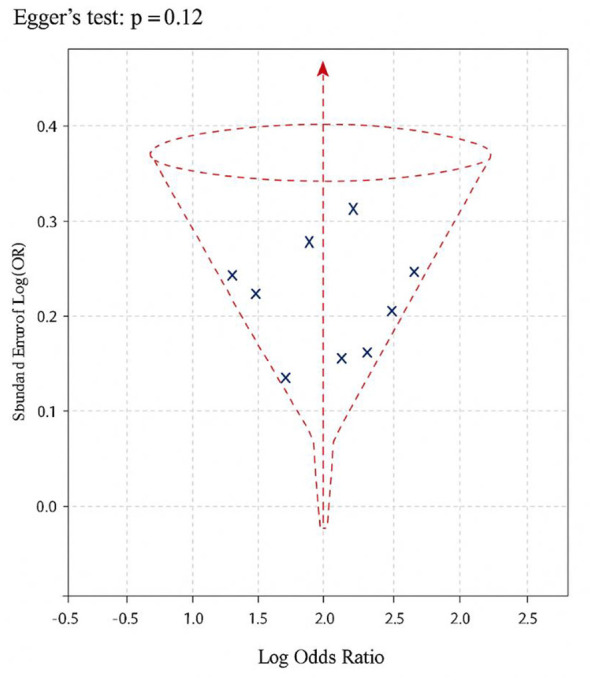
Funnel plot of studies evaluating BPA exposure and CPP.

Visual examination of the funnel plot (Figure 3) revealed a largely symmetrical distribution of effect estimates, indicating minimal publication bias among the included studies. Although a few smaller studies tended to report higher odds ratios, the overall shape of the funnel was balanced around the pooled effect size, suggesting that any observed asymmetry likely reflects random variation or differences in study precision rather than systematic bias.

Following visual inspection, Egger's regression test showed no statistically significant small-study effects (*p* = 0.12), reinforcing the visual impression of limited publication bias. The symmetry observed supports the reliability of the meta-analysis findings, suggesting that the association between BPA exposure and precocious puberty is not substantially influenced by selective reporting or study size variation.

### 3.10 Certainty of evidence

The certainty of evidence for the association between bisphenol A (BPA) exposure and precocious puberty was assessed using the GRADE framework. Given that all included studies were observational (case–control and cross-sectional; Table 2), the baseline certainty was considered low. Upgrades were applied for consistent effect direction, demonstration of a dose–response relationship, and biological plausibility supported by endocrine-disrupting mechanisms. Downgrades were applied for moderate heterogeneity and imprecision in certain subgroup analyses.

Evidence for a dose–response relationship (25, 27, 29) was rated as high certainty, reflecting reproducible trends across multiple studies and BPA measurement methods. Female-specific effects (22–27, 29) were rated moderate certainty, supported by consistent associations and plausible hormonal mechanisms. Male-specific effects (27, 28, 30) were rated low certainty due to small sample sizes and inconsistent findings. Evidence for geographic variation (22–30) was rated moderate certainty, as associations were largely consistent across Asian and non-Asian populations despite some methodological heterogeneity. Overall, the GRADE assessment indicates moderate to high confidence in the association of BPA exposure with early pubertal onset in girls, with supportive but lower certainty evidence for dose-response and male-specific outcomes.

## 4 Discussion

This systematic review and meta-analysis synthesized evidence from nine observational studies conducted across Asia, Europe, and Africa to evaluate the association between postnatal bisphenol A (BPA) exposure and early pubertal outcomes, including central precocious puberty (CPP) and premature thelarche (PT). Higher BPA exposure was observationally associated with increased odds of early pubertal onset (pooled OR = 1.95, 95% CI: 1.60–2.50). Associations were more pronounced in case–control studies and in those using high-sensitivity analytical methods such as LC–MS/MS, underscoring the influence of exposure assessment quality. Subgroup analyses by geographic region and study quality rating further suggested that studies conducted in East Asia and those with high-quality BPA measurements reported stronger associations than studies conducted in other regions or with lower quality ratings. Dose–response analyses from three studies (25, 27, 29, 31–33) suggested an approximate 14% increase in odds per 1 μg/L BPA increment, although this estimate is based on limited data and should be interpreted cautiously. Potential physiological mechanisms linking BPA exposure to early pubertal onset include estrogenic activity through nuclear estrogen receptor binding, modulation of gonadotropin levels, and alterations in steroid metabolism, as observed in several included studies (22, 24, 25), which provides biological plausibility for the observed associations. Specifically, BPA may accelerate puberty by binding to estrogen receptors, altering the hypothalamic-pituitary-gonadal axis, influencing steroidogenesis, and affecting gonadotropin secretion, as suggested by studies showing correlations between BPA levels and basal/peak LH, estradiol, and bone age (22–25, 27).

Although these findings do not have immediate clinical or regulatory implications, they are relevant from a public health perspective because BPA exposure is widespread, occurs during sensitive developmental periods, and is potentially modifiable. While causality cannot be inferred, the observed associations support continued exposure surveillance and precautionary risk communication and contribute to the broader evidence base considered by regulatory agencies, without warranting immediate policy or clinical practice changes. Furthermore, BMI may act as both a confounder and an effect modifier, and subgroup analyses indicated that associations might be stronger among overweight/obese girls (25), highlighting the importance of stratifying by weight status when interpreting the relationship between BPA exposure and early puberty. The predominance of female participants (1,420 girls vs. 40 boys) and reliance on single-time-point BPA measurements further restrict generalizability and limit the ability to assess sex-specific effects, with the small male sample size warranting caution in interpretation.

### 4.1 Limitations

Several limitations should be acknowledged. This review is limited by the absence of cohort studies, as most included studies were case–control or cross-sectional, which restricts causal inference. Additionally, the relatively small and institution-specific populations may limit the generalizability of our findings to broader populations, particularly for male participants, for whom evidence is sparse. The evidence is derived exclusively from observational studies, limiting causal inference. Moderate heterogeneity was observed (I^2^ = 65%) and was only partially explained by study design and analytical methods; therefore, the pooled estimate should be interpreted as an average effect, and a prediction interval would likely be wide. Several studies, including Chen et al. (27), reported wide confidence intervals, reflecting imprecision. In addition, BPA exposure was often measured at or near diagnosis in case–control studies, while cross-sectional studies (28, 30) assessed exposure and outcome concurrently, limiting confirmation of temporal relationships. The predominance of female participants (1,420 girls vs. 40 boys) and reliance on single-time-point BPA measurements further restrict generalizability and limit the ability to assess sex-specific effects, with the small male sample size warranting caution in interpretation.

### 4.2 Future research recommendations

Future research should prioritize longitudinal cohort studies with repeated BPA measurements using standardized, high-precision analytical methods. Harmonized definitions of pubertal outcomes and adequate representation of boys are needed to clarify sex-specific effects and exposure–response relationships. Continued biomonitoring of BPA exposure in children will be important to inform risk assessment and guide evidence-based prevention strategies.

## 5 Conclusion

In conclusion, this systematic review and meta-analysis demonstrates a consistent observational association between postnatal bisphenol A (BPA) exposure and early pubertal onset, particularly among girls. Although a dose–response trend and biological plausibility were observed, the evidence does not establish causality and is based on a limited number of studies. From a public health perspective, these findings support continued exposure monitoring, biomonitoring programs, and well-designed longitudinal research rather than immediate clinical or regulatory action. Improved characterization of exposure–response relationships, sex-specific effects, and critical exposure windows will be essential for informing future prevention strategies and contextualizing early pubertal development within an environmental health framework.

## Data Availability

The original contributions presented in the study are included in the article/supplementary material, further inquiries can be directed to the corresponding author.
