# Correction: Postnatal Bisphenol A exposure and risk of precocious puberty in children: updated systematic review and meta-analysis

**DOI:** 10.3389/fpubh.2026.1847565

**Published:** 2026-04-24

**Authors:** Bassam Bin-Abbas, Mosleh Ali Jabari

**Affiliations:** 1 Department of Pediatrics, King Faisal Specialist Hospital and Research Center, Riyadh, Saudi Arabia; 2 Department of Pediatrics and Endocrinology, Imam Mohammad Ibn Saud Islamic University, Riyadh, Saudi Arabia

**Keywords:** Bisphenol A, children, endocrine disruptors, meta-analysis, precocious puberty, pubertal timing

An incorrect number was provided for Deanship of Scientific Research at Imam Mohammad Ibn Saud Islamic University (IMSU). The correct number is grant number IMSU-DDRSP2601.

The original version of this article has been updated.

