# Supplementary material for: Postnatal Bisphenol A exposure and risk of precocious puberty in children: updated systematic review and meta-analysis
Source: Front Public Health. 2026 Apr 1;14:1776405. doi: 10.3389/fpubh.2026.1776405 (PMC13079639; doi:10.3389/fpubh.2026.1776405)
Supplement: Supplementary file 1 [file Table_1.docx]

Supplementary Table 1. Full reproducible search strategies for all databases for postnatal BPA exposure and central precocious puberty.

|  |  |  |  |
| --- | --- | --- | --- |
| **Database** | **Date of Search** | **Search Strategy / Keywords** | **Filters Applied** |
| **PubMed/MEDLINE** | Jan 2000 – Dec 2024 | ("Child"[Mesh] OR "Adolescent"[Mesh] OR pediatric OR girl OR boy OR youth OR teenager) AND ("Phenols"[Mesh] OR "Endocrine Disruptors"[Mesh] OR "Bisphenol A" OR BPA OR "environmental phenol" OR plasticizer) AND ("Puberty, Precocious"[Mesh] OR "Sexual Precocity"[Mesh] OR "precocious puberty" OR "early puberty" OR "premature gonadarche" OR thelarche OR adrenarche OR gonadarche OR "pubertal onset" OR "pubertal development" OR "sexual maturation") AND ("Puberty"[Mesh] OR "Menarche"[Mesh] OR "Gonadarche"[Mesh] OR "pubertal stage" OR "Tanner stage" OR "pubertal transition" OR "adolescent development" OR "sexual development") | No language or publication status restrictions |
| **Embase** | Jan 2000 – Dec 2024 | (‘child’/exp OR ‘adolescent’/exp OR pediatric OR girl OR boy OR youth OR teenager) AND (‘phenol’/exp OR ‘endocrine disruptor’/exp OR ‘bisphenol a’ OR BPA OR ‘environmental phenol’ OR plasticizer) AND (‘precocious puberty’/exp OR ‘sexual precocity’ OR ‘precocious puberty’ OR ‘early puberty’ OR ‘premature gonadarche’ OR thelarche OR adrenarche OR gonadarche OR ‘pubertal onset’ OR ‘pubertal development’ OR ‘sexual maturation’) AND (‘puberty’/exp OR menarche OR gonadarche OR ‘pubertal stage’ OR ‘tanner stage’ OR ‘pubertal transition’ OR ‘adolescent development’ OR ‘sexual development’) | No language or publication status restrictions |
| **Web of Science Core Collection** | Jan 2000 – Dec 2024 | TS=(child OR adolescent OR pediatric OR girl OR boy OR youth OR teenager) AND TS=(“bisphenol A” OR BPA OR “environmental phenol” OR plasticizer) AND TS=(“precocious puberty” OR “early puberty” OR “premature gonadarche” OR thelarche OR adrenarche OR gonadarche OR “pubertal onset” OR “pubertal development” OR “sexual maturation”) | No language restrictions |
| **Scopus** | Jan 2000 – Dec 2024 | TITLE-ABS-KEY (child OR adolescent OR pediatric OR girl OR boy OR youth OR teenager) AND TITLE-ABS-KEY (“bisphenol A” OR BPA OR “environmental phenol” OR plasticizer) AND TITLE-ABS-KEY (“precocious puberty” OR “early puberty” OR “premature gonadarche” OR thelarche OR adrenarche OR gonadarche OR “pubertal onset” OR “pubertal development” OR “sexual maturation”) | No language restrictions |
| **Cochrane Central Register of Controlled Trials** | Jan 2000 – Dec 2024 | MeSH descriptors: [Child], [Adolescent], [Phenols], [Endocrine Disruptors], [Puberty, Precocious] AND free text: pediatric OR girl OR boy OR youth OR teenager OR BPA OR “Bisphenol A” OR “precocious puberty” | No filters |
| **PsycINFO** | Jan 2000 – Dec 2024 | (DE “Child” OR DE “Adolescent”) AND (DE “Endocrine Disruptors” OR “Bisphenol A” OR BPA OR “environmental phenol”) AND (DE “Puberty, Precocious” OR “precocious puberty” OR “early puberty” OR thelarche OR adrenarche OR gonadarche) | No language restrictions |
| **ClinicalTrials.gov** | Jan 2000 – Dec 2024 | “Bisphenol A” OR BPA AND “precocious puberty” | All recruitment statuses included |
| **WHO ICTRP** | Jan 2000 – Dec 2024 | “Bisphenol A” OR BPA AND “precocious puberty” | All recruitment statuses included |
| **ProQuest Dissertations & Theses Global** | Jan 2000 – Dec 2024 | “Bisphenol A” OR BPA AND “precocious puberty” | No language restrictions |

PICOS: Population, Intervention, Comparison, Outcome, Timing; CPP: Central Precocious Puberty; BPA: Bisphenol A; LH: Luteinizing Hormone; GnRH: Gonadotropin-Releasing Hormone. This supplementary table shows the full reproducible search strategies applied across multiple databases, including PubMed/MEDLINE, Embase, Web of Science Core Collection, Scopus, Cochrane Central Register of Controlled Trials, PsycINFO, ClinicalTrials.gov, WHO International Clinical Trials Registry Platform, and ProQuest Dissertations & Theses Global, for identifying studies on postnatal BPA exposure and central precocious puberty. The search strategies combined controlled vocabulary (MeSH and Emtree terms) with free-text keywords mapped to the PICOS framework, using Boolean operators adapted for each database, without restrictions on language or publication status.
